# Managing Minds at Work: development of a digital line manager training programme

**DOI:** 10.1093/eurpub/ckac130.209

**Published:** 2022-10-25

**Authors:** H Blake, B Vaughan, C Bartle, J Yarker, F Munir, S Marwaha, S Russell, C Meyer, J Hassard, L Thomson

**Affiliations:** 1 School of Health Sciences, University of Nottingham, Nottingham, UK; 2 NIHR Nottingham Biomedical Research Centre, University of Nottingham, Nottingham, UK; 3 Institute of Mental Health, Nottinghamshire Healthcare NHS Trust, Nottingham, UK; 4 Birkbeck, University of London, London, UK; 5 School of Sport, Exercise & Health Sciences, Loughborough University, Loughborough, UK; 6 Institute of Mental Health, University of Birmingham, Birmingham, UK; 7 Thrive at Work, West Midlands Combined Authority, Birmingham, UK; 8 Executive Office, Warwick University, Warwick, UK; 9 School of Medicine, University of Nottingham, Nottingham, UK

## Abstract

**Background:**

Mental ill health is the leading cause of sickness absence with high economic burden. Workplace interventions aimed at supporting employers with prevention of mental ill-health in the workforce are urgently required. Managing Minds at Work (MMW) is a digital intervention targeting support for line managers in any work setting to promote better mental health at work through a preventative approach.

**Objectives:**

To describe the design and development of the MMW digital training programme, prior to feasibility testing. We adopted a collaborative participatory design involving co-design (users as partners) and principles of user-centred design (pilot and usability testing). Agile methodology was used to co-create intervention content with a stakeholder community of practice. Development processes were mapped to core elements of the Medical Research Council (MRC) framework for developing and evaluating complex interventions.

**Results:**

The program covers five broad areas: (i) promoting self-care techniques among line managers; (ii) designing work to prevent work-related stress; (iii) management competencies to prevent and reduce stress; (iv) having conversations with employees about mental health; (v) building a psychologically safe work environment. Pilot and usability testing (n = 37 surveys) aligned with the Technology Acceptance Model (TAM) demonstrated that MMW was perceived to be useful, relevant, and easy to use by managers across sectors, organization types and sizes. We identified positive impacts on manager attitudes and behavioural intentions related to preventing mental ill-health and promoting good mental well-being at work.

**Conclusions:**

MMW is a digital training programme for line managers that has been co-created using rigorous development processes and aims to support employers with primary prevention in mental health. The next step is to explore the feasibility and acceptability of this intervention with line managers in diverse employment settings.

**Key messages:**

- We used co-creation and participatory design to create Managing Minds at Work - a new digital intervention to support line managers in preventing mental ill-health at work.
- The next step is to explore the feasibility and acceptability of this intervention with line managers in diverse employment settings.

